# Evaluation of the Strength at Home Group Intervention for Intimate Partner Violence in the Veterans Affairs Health System

**DOI:** 10.1001/jamanetworkopen.2023.2997

**Published:** 2023-03-14

**Authors:** Suzannah K. Creech, Justin K. Benzer, LeAnn Bruce, Casey T. Taft

**Affiliations:** 1 Veterans Affairs Veterans Integrated Services Network 17 Center of Excellence for Research on Returning War Veterans, Waco, Texas; and the Central Texas Veterans Health Care System, Temple; 2 Dell Medical School of the University of Texas at Austin, Austin; 3 Department of Veterans Affairs, Veterans Health Care Administration, Care Management and Social Work, Washington, DC; 4 National Center for PTSD, Veterans Affairs Boston Healthcare System, Boston, Massachusetts; 5 Boston University Chobanian & Avedisian School of Medicine, Boston, Massachusetts

## Abstract

**Importance:**

Intimate partner violence (IPV) is a serious and prevalent public health issue that is interconnected with experiences of trauma, mental and physical health difficulties, and health disparities. Strength at Home (SAH) is a group intervention for persons using IPV in their relationships. Although previous studies have provided evidence of SAH’s effectiveness in reducing IPV, its patient outcomes as implemented within organized health care have not been examined.

**Objective:**

To evaluate patient outcomes from implementation of SAH in the Department of Veterans Affairs (VA) health system.

**Design, Setting, and Participants:**

This quality improvement study evaluated patient outcomes from a national implementation and training program conducted between December 11, 2015, and September 24, 2021. Data were collected as part of treatment and submitted by clinicians at 73 VA health care facilities. Patients were 1754 veterans seeking care aimed at addressing and/or preventing their use of aggression in intimate relationships. They completed 1 pretreatment assessment and 1 follow-up assessment in the immediate weeks after group completion.

**Intervention:**

Strength at Home is a 12-week trauma-informed and cognitive behavioral group intervention to address and prevent the use of IPV in relationships.

**Main Outcomes and Measures:**

Changes in IPV were measured with the Centers for Disease Control and Prevention 2010 National Intimate Partner and Sexual Violence Survey. Changes in posttraumatic stress disorder (PTSD) symptoms were measured with the PTSD Checklist for *DSM-5*, and alcohol misuse was measured with the Alcohol Use Disorders Identification Test.

**Results:**

The study included 1754 participants (mean [SD] age, 44.3 [13.0] years; 1421 men [81%]), of whom 1088 (62%) were involved with the criminal legal system for IPV charges. Analyses indicate that SAH was associated with reductions in use of physical IPV (odds ratio, 3.28; percentage difference from before to after treatment, –0.17 [95% CI, −0.21 to −0.13]) and psychological IPV (odds ratio, 2.73; percentage difference from before to after treatment, –0.23 [95% CI, −0.27 to −0.19]), coercive control behaviors (odds ratio, 3.19; percentage difference from before to after treatment, –0.18 [95% CI, –0.22 to –0.14), PTSD symptoms (mean change, −4.00; 95% CI, 0.90-7.09; Hedges *g* = 0.10), and alcohol misuse (mean change, 2.70; 95% CI, 1.54-3.86; Hedges *g* = 0.24).

**Conclusions and Relevance:**

In this quality improvement study of the patient outcomes after implementation of SAH, results suggested that the program was associated with reductions in IPV behaviors, PTSD symptoms, and alcohol misuse. Results also suggest that IPV intervention in routine health care at VA health care facilities was successful; extension to other organized health care systems could be warranted.

## Key Points

**Question:**

Is the Strength at Home (SAH) intervention associated with reductions in intimate partner violence (IPV) in an implementation evaluation at 73 Department of Veterans Affairs (VA) health care facilities?

**Findings:**

This quality improvement study examined preintervention and postintervention outcomes from 1754 patients who participated in an implementation and training program. Results suggested that SAH was associated with reductions in IPV, posttraumatic stress disorder symptoms, and alcohol misuse.

**Meaning:**

The findings suggest that SAH was associated with improvement in IPV behaviors and associated problems and that IPV intervention was successful as part of routine health care at VA facilities.

## Teaser

This quality improvement study evaluates patient outcomes from implementation of the Strength at Home intervention, a group program for individuals using intimate partner violence in their relationships, in the Veterans Affairs (VA) health system.

## Introduction

Intimate partner violence (IPV) is a serious and prevalent national public health issue, with a lifetime economic burden estimated at $3.6 trillion US dollars in adult survivor effects^1^ and another $55 billion US dollars in child survivor effects.^2^ Lifetime prevalence estimates of IPV in the US suggest that rates range from 37.3% of women and 30.9% of men for the experience of sexual coercion, physical violence, or stalking and 47.1% of women and 47.3% of men for psychological aggression.^3^ Among persons experiencing IPV, consequences include significant and multigenerational physical health, mental health, and social, educational, and occupational difficulties.^4,5,6,7,8^ Psychological trauma and its correlates, posttraumatic stress disorder (PTSD) in particular, are recognized as important factors associated with IPV,^9,10^ raising the concern that IPV and trauma may interact to worsen health and mental health outcomes and exacerbate health disparities.^11,12^

Historically, intervention for IPV has been overseen by the US criminal legal system, with important individual results but little change in the rates of recidivism overall.^13,14,15^ Intervening to address IPV only after someone has come into contact with the criminal legal system limits the accessibility and impact of existing programs.^16^ In addition to being provided in a punitive context, typical IPV intervention programs are costly, often explicitly prohibit consideration of the role of trauma and mental health as factors associated with IPV,^17^ and, most important, lack evidence for effectiveness.^13,14^ An alternative model is to address IPV as a public health issue; from this perspective, health care systems have an increasingly important role in advancing evidence-based solutions.^18^ In recognition of the high prevalence of IPV among veterans,^19^ in 2013, the Department of Veterans Affairs (VA) embarked on a systematic health care transformation to integrate IPV screening, prevention, and treatment into the health care offered to veterans.^20^ A key part of the scaling up of the VA’s Intimate Partner Violence Assistance Program was the implementation of Strength at Home (SAH), an evidence-based and trauma-informed group intervention to address the use of violence in intimate relationships.

Strength at Home was initially funded by the VA and Department of Defense to be a model IPV intervention program for military and veteran populations. Strength at Home uses cognitive behavioral and motivational enhancement approaches within a closed-group format. Delivered over twelve 120-minute sessions, the content of SAH is grounded in the social-information processing model of IPV, which holds that trauma and PTSD bias perception of social information and choice of response in a way that can facilitate the use of aggression.^21^ Strength at Home is typically facilitated in a group setting and is delivered in 4 phases: (1) psychoeducation on IPV and common reactions to trauma, (2) conflict management skills, (3) coping strategies and negative thought patterns, and (4) communication skills.^22^ Veterans must complete practice assignments, including self-monitoring exercises and practice of skills taught in group. The SAH program addresses the influence of trauma exposure and core themes linking trauma to relationship problems and IPV, which is a unique program feature.^22^ An innovative aspect of the program is that participants’ partners are contacted via telephone by VA clinicians and are provided hotline numbers and resources and given the option to create a safety plan, which is an often overlooked gap in other intervention programs addressing IPV.^23^

Strength at Home has demonstrated reductions in psychological and physical IPV in pilot, efficacy, and effectiveness trials.^22,24,25,26,27^ A randomized clinical trial with 135 participants examined the effect of SAH compared with an enhanced treatment as usual waiting list condition. Results indicated significant reductions in physical and psychological IPV as well as in the use of coercive control behaviors after treatment and 3 months later as reported by both veterans and their partners.^24^ A follow-up effectiveness study examined the effect of SAH in the waiting list control group, and results again indicated significant reductions in the use of aggression.^25^ Recent studies have also observed significant reductions in aggression and PTSD symptoms among veterans receiving the program as part of a pilot implementation within the VA health care system,^22^ and the program has shown evidence of effectiveness among a sample of men in a non-VA setting.^26^ Although alcohol misuse is not an explicit target of SAH, its association with IPV is addressed clinically when it is an issue for participants, and effectiveness studies have demonstrated significant reductions in alcohol misuse from before to after treatment.^26^

The primary goal of the present evaluation was to examine changes in the occurrence of IPV, PTSD symptoms, and alcohol misuse among 1754 veterans who received SAH at 73 VA health care facilities during a 6-year national implementation and training program for VA clinicians. It was hypothesized that participation in the SAH program would be associated with reductions in IPV, PTSD symptoms, and alcohol misuse.

## Methods

The institutional review board at VA Boston reviewed the procedures and determined this evaluation of patient-level implementation outcomes did not meet the criteria for human research and was exempt from further institutional review board review. Individual patient data were deidentified. This quality improvement study is a nonrandomized evaluation of implementation outcomes and is not a randomized or experimental trial; therefore, it is reported in accordance with the revised Standards for Quality Improvement Reporting Excellence (SQUIRE) reporting guideline.^28^

### Participants

Patients in this study (N = 1754) were treated by VA clinicians participating in the SAH training program and were drawn from 73 VA health care facilities throughout the US between December 11, 2015, and September 24, 2021. The clinician training program included a 2-day experiential workshop, weekly clinical consultations, and monthly implementation consultations with national experts in SAH.^29^ It also included the systematic collection of program evaluation information, which formed the data set for this study.

Patients in this study completed 1 pretreatment assessment and 1 follow-up assessment in the immediate weeks after group completion. Patients were not required to have any primary diagnosis but to be interested in addressing and/or preventing their use of aggression in intimate relationships. Many were court ordered to receive IPV intervention or were otherwise involved with the criminal legal system. Clinicians used their clinical judgment in determining enrollment but received explicit instruction that almost all veterans referred to the program would be appropriate for SAH. The only exception was in the case of acute need for crisis stabilization, in which case the patient would be welcomed back once stable. Clinicians provided referrals for additional services as needed throughout the program. Baseline demographic and program completion data are presented in Table 1.

**Table 1.** Veteran Demographic and Completion Data

Characteristic	Veterans, No. (%)	
	Full sample (N = 1754)	Subset with posttreatment data (n = 940)^a^
Age, mean (SD), y	44.3 (13.0)	45.1 (13.6)
Sex		
Male	1421 (81)	713 (76)
Female	29 (2)	4 (0.4)
Missing	304 (17)	223 (24)
Court involved		
Yes	1088 (62)	639 (68)
No	641 (37)	291 (31)
Missing	25 (1)	10 (1)
Race		
American Indian or Alaska Native	25 (1)	15 (2)
Asian	20 (1)	11 (1)
Black	449 (26)	241 (26)
Native Hawaiian or Pacific Islander	17 (1)	9 (1)
White		
Hispanic	120 (7)	61 (7)
Non-Hispanic	1028 (59)	557 (59)
Other^b^	59 (3)	30 (3)
Missing	28 (2)	14 (1)
Ethnicity		
Hispanic	194 (11)	102 (11)
Non-Hispanic	1543 (88)	833 (89)
Missing	17 (1)	5 (1)
Service era		
Korea	10 (1)	5 (1)
Vietnam	192 (11)	120 (13)
Persian Gulf	285 (16)	152 (16)
Iraq	500 (29)	239 (25)
Afghanistan	738 (42)	413 (44)
Missing	29 (2)	11 (1)
Sessions attended, mean (SD)	8.6 (3.5)	10.4 (1.6)
Completed ≥9 sessions		
Yes	1190 (68)	869 (92)
No	564 (32)	71 (8)

^a^ Refers to patients who completed at least 1 posttreatment outcome measure.

^b^ Includes patients who self-reported identifying as multiple races or ethnicities, or as “other.”

When patients completed release of information forms to allow hospital staff to conduct a partner outreach telephone call, a clinician attempted to contact partners up to 3 times around the beginning and end of the 12-week group. During these calls, all partners were offered hotline numbers and the chance to make a safety plan, and if they wished, they also reported measures of IPV they experienced by their partner, which were intended for use by clinicians to help understand the scope of the IPV reported and any differences in perspectives between partners and veterans. Clinicians reported that they completed 545 partner telephone calls: 381 partner intake calls and 164 partner posttreatment calls.

### Measures

All patients were seen for a baseline assessment and interview before starting an SAH group, during which they agreed to participate in the program, signed any appropriate release of information forms, and completed baseline self-report measures of IPV, PTSD symptoms, and alcohol misuse. Participants also self-reported their age, gender identity, era of military service, and race and ethnicity during the intake interview. These variables were collected to understand the reach of the program across demographic groups of veterans. After the baseline assessment, participants were seen for up to 12 sessions of the SAH group. The same measures, plus a posttreatment satisfaction measure, were completed after session 12. Attendance in 9 of 12 sessions (75%) was considered completion, and those who attended at least 9 sessions also received a certificate of completion. Clinicians submitted deidentified scale scores and patient demographic characteristic information to program evaluation staff as part of the training program.

Intimate partner violence was measured using questions from the Centers for Disease Control and Prevention 2010 National Intimate Partner and Sexual Violence Survey,^30^ which included 30 items assessing 4 subtypes of IPV behavior: (1) psychological aggression (eg, “I acted very angry towards my partner in a way that seemed dangerous”), (2) coercive control (eg, “I tried to keep my partner from seeing or talking to their family or friends”), (3) reproductive control (eg, “I tried to get my partner pregnant when they did not want to get pregnant”), and (4) physical aggression (eg, “I slapped my partner”). At 2 time points (before and after treatment), veterans reported whether they had engaged in each IPV behavior in the past 3 months on a dichotomous scale, with 0 indicating no and 1 indicating yes. Prevalence scores were calculated by summing the number of positive responses for each subtype of IPV and converting the sum into a dichotomous variable representing the presence or absence of each type of IPV in the past 3 months.^31^ Summary scores representing the total number of subtypes of IPV (ie, psychological, coercive, reproductive, and physical) were also computed (possible range, 0-4).

Posttraumatic stress disorder symptoms were measured with the 20-item PTSD Checklist for the *Diagnostic and Statistical Manual of Mental Disorders* (Fifth Edition) (PCL-5).^32^ Participants indicated how often they were bothered by each symptom in the past month on a 5-point Likert scale (0 = not at all and 4 = extremely), and participants reported symptoms based on “a very stressful experience.” Items were summed, with higher scores reflecting more severe symptoms. A cut score of 33 is suggested as indicative of probable PTSD.^33^ The measure evidences good reliability (internal consistency, 0.96; test-retest reliability, 0.84) and discriminant and convergent validity.

The Alcohol Use Disorders Identification Test (AUDIT) was used to assess alcohol misuse over the past year.^34^ The AUDIT is a 10-item screener with 3 questions on the amount and frequency of drinking, 3 questions on alcohol dependence, and 4 questions on problems caused by alcohol; responses (on a 0- to 4-point scale) were summed, with higher scores reflecting greater levels of alcohol misuse. A cut score of 8 is suggested as indicative of problem or hazardous drinking.^35^ The AUDIT has a reported median reliability coefficient of 0.83 and adequate construct and criterion-related validity.^36^

### Statistical Analysis

Data analysis compared pretreatment and posttreatment IPV, PCL-5, and AUDIT scores. The sample included all participants who completed at least 1 in-person SAH group session and had nonmissing IPV, PCL-5, and AUDIT scores. The pattern of missing data was predominantly missing posttreatment data (eTable in Supplement 1). Regarding missing data, 578 of 1754 individuals (33%) had complete data, 890 of 1754 participants (51%) had at least 1 missing posttreatment variable, and 809 of 1754 participants (46%) had no posttreatment data. Multiple imputation was used to estimate missing posttreatment data using pretreatment scores, as well as demographic characteristics and lifetime IPV variables that correlated significantly with the observed scores and/or the state of being missing. Multiple imputation was conducted with SAS, version 9.4 (SAS Institute Inc), with the Markov chain Monte Carlo method for the AUDIT and PCL-5 measures and the fully conditional specification method for the dichotomous IPV measures. We specified 20 imputations and 100 burn-in iterations. The difference between pretreatment and posttreatment measures were estimated using the McNemar χ^2^ tests for binary variables and 1-sided *t* tests for the interval and count variables. Effect sizes were calculated as odds ratios (ORs) for the binary variables and as Hedges *g* for the interval and count variables. Significance was set using the Benjamini-Hochberg method with a false discovery rate of 5%.^37^

## Results

A total of 1754 veterans (mean [SD] age, 44.3 [13.0] years; 1421 men [81%]) completed an intake for the SAH program (see Table 1 for complete demographic characteristics); these veterans were aged 21 to 86 years, with 449 (26%) self-identifying as Black, 194 (11%) as Hispanic, 1028 (59%) as non-Hispanic White, and 121 (7%) identifying as another race or ethnicity (including American Indian or Alaska Native, Asian, Native Hawaiian or Pacific Islander, multiracial, or other). The sample included veterans who were involved with the criminal legal system for IPV charges (1088 [62%]) and veterans who predominantly (1238 [71%]) served in the recent US wars in Iraq and Afghanistan.

Table 2 provides changes in IPV prevalence. There were significant reductions from before to after treatment in the proportion of the sample of veterans who reported physical IPV (percentage change, −0.17; 95% CI, −0.21 to −0.13), psychological IPV (percentage change, −0.23; 95% CI, −0.27 to −0.19), coercive control behaviors (percentage change, −0.18; 95% CI −0.22 to −0.14), and the presence of any IPV (percentage change −0.23; 95% CI, −0.28 to −0.19). The frequency of reproductive control was low (44 of 1754 [3%] at the pretreatment period) and changes were not significant (percentage change, −0.01; 95% CI −0.01 to 0.02). Odds ratios indicated that for every person who engaged in any IPV after SAH, 2.65 people did not, with ORs ranging from 3.28 for physical IPV to 2.73 for psychological IPV and 3.19 for coercive control. The OR for reproductive control was high, due in part to the low base rate, but the difference was not significant.

**Table 2.** Association of SAH Program With IPV Prevalence

IPV subtype	Veterans, No. (%) (N = 1754)		Odds ratio^a^	Difference (95% CI), %^b^
	Pretreatment	Posttreatment		
Physical IPV	601 (34)	307 (18)	3.28	−0.17 (−0.21 to −0.13)^c^
Psychological IPV	964 (55)	561 (32)	2.73	−0.23 (−0.27 to −0.19)^c^
Coercive control	776 (44)	456 (26)	3.19	−0.18 (−0.22 to −0.14)^c^
Reproductive control	44 (3)	59 (3)	17.27	−0.01 (−0.01 to 0.02)
Any IPV	1102 (63)	693 (40)	2.65	−0.23 (−0.28 to −0.19)^c^

Abbreviations: IPV, intimate partner violence; SAH, Strength at Home.

^a^ The odds ratio is the odds of event in the group where IPV is present at baseline divided by the odds of the event in the group where IPV is not present at baseline.

^b^ Significance of the difference scores evaluated using the Benjamani-Hochberg score.

^c^ Significant at *P* < .05.

Table 3 presents the changes in the sum of the number of IPV subtypes, PTSD symptoms, and alcohol misuse. Significant improvements were observed for all 3 outcomes. Effect sizes were small for PTSD symptoms (mean change, −4.00; 95% CI, 0.90-7.09; Hedges *g* = 0.10) and alcohol misuse (mean change, 2.70; 95% CI, 1.54-3.86; Hedges *g* = 0.24) and medium for the number of IPV subtypes reported (mean change, 0.48; 95% CI, 0.47-0.68; Hedges *g* = 0.57).

**Table 3.** Association of SAH With Number of IPV Subtypes Reported, PTSD Symptoms, and Alcohol Misuse Among 1754 Patients

Variable	Pretreatment, mean	Posttreatment, mean	Hedges *g*	Difference (95% CI)^a^
IPV subtypes	1.36	0.79	0.57	0.48 (0.47-0.68)^b^
PTSD symptoms	39.23	35.24	0.10	4.00 (0.90-7.09)^b^
Alcohol misuse	6.81	4.11	0.24	2.70 (1.54-3.86)^b^

Abbreviations: IPV, intimate partner violence; PTSD, posttraumatic stress disorder; SAH, Strength at Home.

^a^ Significance of the difference scores evaluated using the Benjamani-Hochberg score.

^b^ Significant at *P* < .05.

We conducted several additional analyses to assess whether court referrals, race and ethnicity, and age were associated with both the prevalence of IPV and the likelihood of completing SAH. None of the variables were associated with the prevalence of IPV. Only court referrals were significantly associated with the likelihood of completing SAH. The OR was modest (1.53; 95% CI, 1.38-1.71), indicating that the veterans mandated to participate in the program were more likely to complete it. However, the modest effect size indicates that being mandated to participate in the program was not strongly associated with graduation from the program.

## Discussion

In this article, we report implementation outcomes from a sample of veterans who received SAH during a 6-year national implementation and training program for VA clinicians. Results from 1754 veterans indicate that SAH was associated with significant pretreatment to posttreatment reductions in the proportion of veterans who reported using physical, psychological, and coercive control IPV toward a partner and in the number of types of IPV used. The effect sizes for these changes were medium. In addition, small but significant reductions in PTSD symptoms and alcohol misuse from before to after treatment also suggest that the program may be helpful in reducing PTSD-associated distress and symptoms. There was no change in reproductive control behaviors; however, there was little opportunity to examine changes in these behaviors due to the low prevalence of this type of IPV at baseline (3% of the sample).

A common criticism of IPV programs is a high attrition rate, even among veterans mandated to participate.^16^ In this study, court involvement was modestly associated with completion rates, and the noncompletion rate (attending <9 sessions) was 32%. This rate is lower than the 37.8% to 50.8% overall attrition rates reported in meta-analyses^38,39^ of the factors associated with attrition in community IPV intervention programs, which are typically comprised entirely of court-mandated participants who are more likely to complete the intervention. The lower noncompletion rate observed in the present study is notable considering that 37% of the sample participants (641 of 1754) were self- or clinician-referred, and they were a clinical sample not excluded for any psychiatric or substance use disorder. The rate is also notable because 62% of the sample participants were court-involved veterans, a group known to have high rates of substance use and psychiatric difficulties and to experience significant barriers to continuous treatment, such as homelessness.^40,41^

Partner outreach telephone calls to address the unmet safety planning and mental health needs of partners who have experienced IPV are an important emphasis of the SAH program and receive considerable attention in the training program. Data from the present study indicated that 31% of partners (545 of 1754) were successfully reached but that the data were limited with respect to the content of the calls as well as reasons for nonresponse. Future research could examine how to improve rates of completed calls and safety plans made by examining barriers and facilitators to successful partner outreach from the perspectives of veterans, partners, and clinicians.

### Strengths and Limitations

The strengths of this study include the large sample size, diverse veteran subgroups, numerous sites, and routine clinical settings and patients. Despite these strengths, results must be considered against several important limitations. First, because the data were obtained from clinical practice, there was no comparison group or follow-up data to assess whether gains were maintained over time. Future research on the sustainability of SAH is needed to assess whether intervention effectiveness is maintained over time. Another potential limitation is the exclusive use of self-report measures, which raises concerns of self-reporting bias, including underreporting of IPV, but also alleviates concerns of clinician bias. Mitigating this concern is prior work showing that, unlike data from civilians, veterans disclose a greater amount of their own use of IPV compared with what their partners report about them.^42^ In addition, the PTSD^33^ and alcohol use^36^ measures used have also demonstrated excellent construct and criterion validity with other measures. Finally, the set of patients in this study was not drawn for representativeness of the veteran population; specifically, results may not be generalizable to women. We also cannot use these data to examine changes in the proportion of veterans engaging in IPV at a population level. The data available were limited with respect to examining factors associated with noncompletion, which should be explored in future analyses.

## Conclusions

Intimate partner violence is a serious, prevalent, and costly public health problem that has traditionally been stigmatized and most often addressed within the criminal legal system. The recent effort to implement access to comprehensive IPV programming at every VA hospital in the country is an alternative model for addressing violence as a public health issue. Data from this study suggest that participation in the SAH program delivered within routine care at VA facilities was associated with reductions in IPV, PTSD symptoms, and alcohol misuse. These results suggest that IPV intervention as part of health care at VA facilities was successful and that its extension to other organized health care systems may be warranted.

## Notes

Supplement 1. **eTable.** Missing Data Patterns

Supplement 2.
Data Sharing Statement
