# Supplementary material for: Evaluation of the Strength at Home Group Intervention for Intimate Partner Violence in the Veterans Affairs Health System
Source: JAMA Netw Open. 2023 Mar 14;6(3):e232997. doi: 10.1001/jamanetworkopen.2023.2997 (PMC10015307; doi:10.1001/jamanetworkopen.2023.2997)
Supplement: Supplement 1. — eTable. Missing Data Patterns [file jamanetwopen-e232997-s001.pdf]

Supplementary Online Content

Creech SK, Benzer JK, Bruce L, Taft CT. Evaluation of the Strength at Home group intervention for intimate partner violence in the Veterans Affairs health system. *JAMA Netw Open.* 2023;6(3):e232997. doi:10.1001/jamanetworkopen.2023.2997

eTable. Missing Data Patterns

This supplementary material has been provided by the authors to give readers additional information about their work.

© 2023 Creech SK et al. *JAMA Network Open.*

eTable. Missing Data Patterns

Lifetime IPV					Demographics and Service Use							
Grp	COER	PHYS	PSY	REPR	WHIT	BLCK	SAT	AGE	FEML	COURT	HISPN	#Sess
1	X	X	X	X	X	X	X	X	X	X	X	X
2	X	X	X	X	X	X	X	X	X	X	X	X
3	X	X	X	X	X	X	X	X	X	X	X	X
4	X	X	X	X	X	X	X	X	X	X	X	X
5	X	X	X	X	X	X	X	X	X	X	X	X
6	X	X	X	X	X	X	X	X	X	X	X	X
7	X	X	X	X	X	X	X	X	X	X	X	X
8	X	X	X	X	X	X	X	X	X	X	X	X
9	X	X	X	X	X	X	X	X	X	X	X	X
10	X	X	X	X	X	X	X	X	X	X	X	X
11	X	X	X	X	X	X	X	X	X	X	X	X
12	X	X	X	X	X	X	X	X	X	X	X	X
13	X	X	X	X	X	X	X	X	X	X	X	X
14	X	X	X	X	X	X	X	X	X	X	X	X
15	X	X	X	X	X	X	X	X	X	X	X	X
16	X	X	X	X	X	X	X	X	X	X	X	X
17	X	X	X	X	X	X	X	X	X	X	.	X
18	X	X	X	X	X	X	X	X	X	.	X	X
19	X	X	X	X	X	X	X	X	X	.	X	X
20	X	X	X	X	X	X	X	X	.	X	X	X
21	X	X	X	X	X	X	X	X	.	X	X	X
22	X	X	X	X	X	X	X	X	.	X	X	X
23	X	X	X	X	X	X	X	X	.	X	X	X
24	X	X	X	X	X	X	X	X	.	X	X	X
25	X	X	X	X	X	X	X	X	.	X	X	X
26	X	X	X	X	X	X	X	X	.	.	X	X
27	X	X	X	X	X	X	X	.	X	X	X	X
28	X	X	X	X	X	X	X	.	.	X	X	X
29	X	X	X	X	X	X	.	X	X	X	X	X
30	X	X	X	X	X	X	.	X	X	X	X	X
31	X	X	X	X	X	X	.	X	X	X	X	X
32	X	X	X	X	X	X	.	X	X	X	X	X
33	X	X	X	X	X	X	.	X	X	X	X	X
34	X	X	X	X	X	X	.	X	X	X	X	X

© 2023 Creech SK et al. *JAMA Network Open.*

35	X	X	X	X	X	X	.	X	X	X	X	X
36	X	X	X	X	X	X	.	X	X	X	X	X
37	X	X	X	X	X	X	.	X	X	X	X	X
38	X	X	X	X	X	X	.	X	X	X	X	X
39	X	X	X	X	X	X	.	X	X	X	X	X
40	X	X	X	X	X	X	.	X	X	X	X	X
41	X	X	X	X	X	X	.	X	X	X	X	X
42	X	X	X	X	X	X	.	X	X	X	.	X
43	X	X	X	X	X	X	.	X	X	X	.	X
44	X	X	X	X	X	X	.	X	X	.	X	X
45	X	X	X	X	X	X	.	X	X	.	.	X
46	X	X	X	X	X	X	.	X	.	X	X	X
47	X	X	X	X	X	X	.	X	.	X	X	X
48	X	X	X	X	X	X	.	X	.	X	X	X
49	X	X	X	X	X	X	.	X	.	.	X	X
50	X	X	X	X	X	X	.	.	X	X	X	X
51	X	X	X	X	X	X	.	.	.	X	X	X
52	X	X	X	X	X	X	.	.	.	.	X	X
53	X	X	X	X	.	.	X	X	X	X	X	X
54	X	X	X	X	.	.	X	X	X	X	X	X
55	X	X	X	X	.	.	X	X	.	X	X	X
56	X	X	X	X	.	.	X	X	.	X	X	X
57	X	X	X	X	.	.	X	.	.	.	.	X
58	X	X	X	X	.	.	X	.	.	.	.	X
59	X	X	X	X	.	.	.	X	X	X	X	X
60	X	X	X	X	.	.	.	X	X	X	X	X
61	X	X	X	X	.	.	.	X	X	X	X	X
62	X	X	X	X	.	.	.	X	X	X	.	X
63	X	X	X	X	.	.	.	X	.	X	X	X
64	X	X	X	X	.	.	.	.	X	X	X	X
65	X	X	X	X	.	.	.	.	X	.	X	X
66	X	X	X	X	.	.	.	.	.	X	.	X
67	X	X	X	X	.	.	.	.	.	.	.	X
68	X	X	X	.	X	X	X	X	X	X	X	X
69	X	X	X	.	X	X	X	X	.	X	X	X
70	X	X	X	.	X	X	.	X	X	X	X	X
71	X	X	X	.	X	X	.	X	X	X	X	X
72	X	X	X	.	X	X	.	X	X	X	X	X
73	X	X	.	X	X	X	.	X	X	X	X	X

© 2023 Creech SK et al. *JAMA Network Open.*

74	X	X	.	X	X	X	.	X	X	X	.	X
75	X	X	.	X	X	X	.	X	.	X	X	X
76	X	.	X	X	X	X	X	X	X	X	X	X
77	X	.	X	X	X	X	.	X	X	X	X	X
78	X	.	X	X	X	X	.	X	X	X	X	X
79	X	.	X	X	X	X	.	X	X	X	X	X
80	X	.	X	X	X	X	.	X	X	X	X	X
81	X	.	X	.	X	X	X	.	.	X	X	X
82	X	.	X	.	X	X	.	X	X	.	X	X
83	X	.	.	X	X	X	.	X	X	X	X	X
84	.	X	X	X	X	X	X	X	X	X	X	X
85	.	X	X	X	X	X	X	X	X	X	X	X
86	.	X	X	X	X	X	.	X	X	X	X	X
87	.	X	X	X	X	X	.	X	X	X	X	X
88	.	X	.	X	X	X	.	X	X	X	X	X
89	.	X	.	X	X	X	.	X	X	X	X	X
90	.	X	.	X	X	X	.	X	X	X	X	X
91	.	X	.	.	X	X	.	X	X	X	X	X
92	.	.	X	.	X	X	.	X	X	X	X	X
93	.	.	.	.	X	X	X	X	X	X	X	X
94	.	.	.	.	X	X	X	.	.	.	X	X
95	.	.	.	.	X	X	.	X	X	X	X	X
96	.	.	.	.	X	X	.	X	X	X	X	X
97	.	.	.	.	X	X	.	X	X	X	X	X
98	.	.	.	.	X	X	.	X	X	X	X	X

	Time 1 Outcomes						Time 2 Outcomes					
Grp	AUD	PCL	COER	PHYS	PSY	REPR	AUD	PCL	COER	PHYS	PSY	REPR
1	X	X	X	X	X	X	X	X	X	X	X	X
2	X	X	X	X	X	X	X	X	X	X	X	.
3	X	X	X	X	X	X	X	X	X	X	.	X
4	X	X	X	X	X	X	X	X	X	.	X	X
5	X	X	X	X	X	X	X	X	.	X	X	X
6	X	X	X	X	X	X	X	X	.	.	.	.
7	X	X	X	X	X	.	X	X	X	X	X	X
8	X	X	X	.	X	X	X	X	X	X	X	X
9	X	X	.	.	.	.	X	X	X	X	X	X

© 2023 Creech SK et al. *JAMA Network Open.*

10	X	X	X	X	X	X	X	.	X	X	X	X
11	X	.	X	X	X	X	X	X	X	X	X	X
12	X	X	X	X	X	X	.	X	X	X	X	X
13	X	X	X	X	X	X	.	.	X	X	X	X
14	X	X	X	X	X	X	.	.	X	.	X	X
15	X	X	X	X	X	X	.	.	.	.	.	.
16	.	X	X	X	X	X	X	X	X	X	X	X
17	X	X	X	X	X	X	X	X	X	X	X	X
18	X	X	X	X	X	X	X	X	X	X	X	X
19	X	X	X	X	X	X	X	X	.	.	.	.
20	X	X	X	X	X	X	X	X	X	X	X	X
21	X	X	X	X	X	X	X	X	.	X	X	X
22	X	X	X	X	X	X	X	.	X	X	X	X
23	X	.	X	X	X	X	X	X	X	X	X	X
24	X	X	X	X	X	X	.	X	X	X	X	X
25	.	.	X	X	X	X	X	X	X	X	X	X
26	X	X	X	X	X	X	X	X	X	X	X	X
27	X	X	X	X	X	X	X	X	X	X	X	X
28	X	X	X	X	X	X	X	X	X	X	X	X
29	X	X	X	X	X	X	X	X	X	X	X	X
30	X	X	X	.	X	X	X	X	X	X	X	X
31	X	X	X	X	X	X	.	X	X	X	X	X
32	X	X	X	X	X	X	.	.	X	X	X	X
33	X	X	X	X	X	X	.	.	.	.	.	.
34	X	X	X	X	.	X	.	.	.	.	.	.
35	X	X	X	.	X	X	.	.	.	.	.	.
36	X	X	X	.	X	.	.	.	.	.	.	.
37	X	X	.	X	X	X	.	.	.	.	.	.
38	X	X	.	.	.	.	.	.	.	.	.	.
39	X	.	X	X	X	X	.	.	.	.	.	.
40	.	X	X	X	X	X	.	.	.	.	.	.
41	.	.	X	X	X	X	.	.	.	.	.	.
42	X	X	X	X	X	X	.	.	X	X	X	X
43	X	X	X	X	X	X	.	.	.	.	.	.
44	X	X	X	X	X	X	.	.	.	.	.	.
45	X	X	X	X	X	X	.	.	.	.	.	.
46	X	X	X	X	X	X	X	X	X	X	X	X
47	X	X	X	X	X	X	.	.	X	X	X	X
48	X	X	X	X	X	X	.	.	.	.	.	.

© 2023 Creech SK et al. *JAMA Network Open.*

49	X	X	X	X	X	X	.	.	.	.	.	.
50	X	X	X	X	X	X	.	.	.	.	.	.
51	X	X	X	X	X	X	.	.	.	.	.	.
52	X	X	X	X	X	X	.	.	.	.	.	.
53	X	X	X	X	X	X	X	X	X	X	X	X
54	X	X	X	X	X	X	X	X	.	X	X	X
55	X	X	X	X	X	X	X	X	X	X	X	X
56	X	X	X	X	X	X	X	.	X	X	X	X
57	X	X	X	X	X	X	.	.	.	.	.	.
58	.	.	X	X	X	X	X	X	X	X	X	X
59	X	X	X	X	X	X	.	.	X	X	X	X
60	X	X	X	X	X	X	.	.	.	.	.	.
61	X	.	X	X	X	X	.	.	.	.	.	.
62	X	X	X	X	X	X	.	.	.	.	.	.
63	X	X	X	X	X	X	.	.	.	.	.	.
64	X	X	X	X	X	X	.	.	X	X	X	X
65	X	X	X	X	X	X	.	.	.	.	.	.
66	X	X	X	X	X	X	.	.	.	.	.	.
67	X	X	X	X	X	X	.	.	X	X	X	X
68	X	X	X	X	X	X	.	.	X	.	X	.
69	X	X	X	X	X	.	X	X	X	X	X	X
70	X	X	X	X	X	X	.	.	.	.	.	.
71	X	X	X	X	X	.	.	.	.	.	.	.
72	X	X	X	X	.	X	.	.	.	.	.	.
73	X	X	X	X	X	X	.	.	.	.	.	.
74	X	X	X	X	X	X	.	.	.	.	.	.
75	X	X	X	X	X	X	.	.	.	.	.	.
76	X	X	X	.	X	X	X	X	X	X	X	X
77	X	X	X	X	X	.	.	.	.	.	.	.
78	X	X	X	.	X	X	.	.	.	.	.	.
79	X	X	.	X	X	X	.	.	.	.	.	.
80	X	.	X	X	X	X	.	.	.	.	.	.
81	X	X	X	.	X	X	X	X	X	X	X	X
82	X	X	X	.	X	.	.	.	.	.	.	.
83	X	X	X	X	X	X	.	.	.	.	.	.
84	X	X	X	X	X	X	X	X	X	X	X	X
85	X	X	.	X	X	X	X	X	X	X	X	X
86	X	X	X	X	X	X	.	.	.	.	.	.
87	X	X	.	X	X	X	.	.	.	.	.	.

© 2023 Creech SK et al. *JAMA Network Open.*

88	X	X	X	X	.	X	.	.	.	.	.	.
89	X	X	X	.	X	X	.	.	.	.	.	.
90	.	.	X	X	X	X	.	.	.	.	.	.
91	X	X	.	X	.	.	.	.	.	.	.	.
92	X	X	.	.	X	.	.	.	.	.	.	.
93	X	X	.	.	.	.	X	X	X	X	X	X
94	X	X	X	X	X	X	X	X	X	X	X	X
95	X	X	X	X	X	X	.	.	.	.	.	.
96	X	X	X	X	X	.	.	.	.	.	.	.
97	X	X	.	.	.	.	.	.	.	.	.	.
98	.	X	X	X	X	X	.	.	.	.	.	.

Note. Missing data were imputed using the following variables: IPV measured as lifetime prevalence, pre-treatment, and post-treatment, the AUDIT-C and PCL scores pre-treatment and post-treatment, and the following demographic and service use auxiliary variables: white race, black race, treatment satisfaction, age, female gender, court-referred, Hispanic ethnicity, number of sessions completed.

© 2023 Creech SK et al. *JAMA Network Open.*
