# Supplementary material for: Evaluation of the Strength at Home Group Intervention for Intimate Partner Violence in the Veterans Affairs Health System
Source: JAMA Netw Open. 2023 Mar 14;6(3):e232997. doi: 10.1001/jamanetworkopen.2023.2997 (PMC10015307; doi:10.1001/jamanetworkopen.2023.2997)
Supplement: Supplement 2. — Data Sharing Statement [file jamanetwopen-e232997-s002.pdf]

Data Sharing Statement

Creech. Evaluation of the Strength at Home Group Intervention for Intimate Partner Violence in the Veterans Affairs Health System. *JAMA Netw Open*. Published March 14, 2023. doi:10.1001/jamanetworkopen.2023.2997

Data
Data available: No

Additional Information
Explanation for why data not available: Pending appropriate institutional approvals summary data can be shared.
